# Do I want to continue being good? Research on the continuity mechanism of organizational citizenship helping behavior

**DOI:** 10.3389/fpsyg.2026.1773921

**Published:** 2026-05-22

**Authors:** Lvyi Zhang, Jiarong Qian, Yunjie Jiang

**Affiliations:** 1 Nanjing Vocational College of Information Technology, Nanjing, China; 2 Business School, Nantong University, Nantong, China; 3 School of Health Policy & Management, Nanjing Medical University, Nanjing, China

**Keywords:** behavior continuity, organizational citizenship helping behavior, perceived coworker support, perceived team-member exchange, social exchange theory

## Abstract

To explore the conditions for the continuity of employees’ interpersonal helping behavior, this paper constructs a dynamic mediated-moderating model of the two phases of organizational citizenship helping behavior, perceived team-member exchange, and perceived coworker support based on social exchange theory. This paper conducts longitudinal research on corporate employees and finally obtains 620 paired data for the two phases. Empirical analyses show that organizational citizenship helping behavior in Phase-1 is significantly and positively associated with organizational citizenship helping behavior in Phase-2, which suggests that organizational citizenship helping behavior tends to be persistent at the time level; perceived team-member exchange is related to stronger helping behavior continuity; and perceived coworker support is associated with stronger helping behavior continuity and fully accounts for the moderating role of perceived team-member exchange in the association between the two-phased helping behavior. This study helps advance the exploration of coworker relationship in organizations, sheds light on how employees internalize perceived interpersonal relationship into an integrated adjustment mechanism, and provides theoretical insights and practical guidelines for corporate managers on how to optimize coworker relationship and employee behavior.

## Introduction

There’s an old Chinese saying, ‘the roses in her hand, the flavor in mine’, suggests that in the process of helping others, one will also gain satisfaction and happiness. In the workplace, such voluntary and altruistic acts toward colleagues fall under the umbrella of organizational citizenship helping behavior (OCB-H), which refers to ‘spontaneous, discretionary behaviors that directly aid colleagues in their work tasks, contributing to team effectiveness beyond formal job requirements’ (Mo et al., 2023). It is also a core dimension of interpersonal organizational citizenship behavior (OCB-I) that contributes to team cohesion and organizational effectiveness beyond formal job requirements (Bergeron et al., 2013). The modern workplace is no longer a stable environment controlled by upper management but a dynamic knowledge and service-oriented environment that requires employees to work in teams to accomplish their tasks with creativity and flexibility (Kabat-Farr and Cortina, 2017), such a context necessitates a harmonious atmosphere and good relationship within the team (Zhang et al., 2024). Social Exchange Theory points out that good relationships are based on the principle of reciprocity, and mutual help between coworkers is undoubtedly one of the ways of reciprocity.

From the perspective of social psychology, helping behavior is a manifestation of the altruistic style of organizational citizenship behavior (Cheung et al., 2018). Extant research on OCB-H has predominantly focused on two static research streams: its antecedents and immediate outcomes. For example, positive emotions have been found to possibly result in a higher frequency of helping behaviors (Chi et al., 2015), and proactive personality is a strong predictor (Yang et al., 2011). Scholars have also linked OCB-H to favorable individual and organizational outcomes, including higher performance appraisal ratings (Triana et al., 2013), career advancement (Rosopa et al., 2013), and enhanced team effectiveness (Xiao et al., 2020; Yung Chou et al., 2014). Empirical studies have also shown that individuals who provide more help to others are more likely to be promoted and rewarded (Rosopa et al., 2013). Most of the existing studies are based on the static perspective, considering helping behavior as an inherent state and exploring the conditions of its occurrence and the resulting changes in individual and organizational states. However, studies rarely pay attention to the dynamics of helping behavior itself and reveal the internal and external reasons why ‘good people (individuals who sustain voluntary prosocial helping behaviors) are willing to continue to do good things’ in the organization. This static perspective neglects the temporal nature of workplace behavior and the role of interpersonal relationship perceptions in shaping behavioral continuity, leaving unanswered a core practical and theoretical question: What conditions drive the persistence of OCB-H in teams?

To address this gap, the present study draws on social exchange theory to examine the continuity mechanism of OCB-H from the perspective of team interpersonal relationship perceptions, and makes three key contributions to the OCB and workplace relationship literature. First, it adopts a dynamic within-person perspective to examine OCB-H persistence across time, moving beyond static cross-sectional designs to account for the temporal attributes of employee behavior—an element highlighted as critical for organizational behavior research by event system theory (Morgeson et al., 2015) and dynamic organizational studies (Chuang et al., 2019). By modeling OCB-H as a temporally sustained behavior, this study responds to calls for more dynamic research on workplace prosocial behavior. Second, it centers on team-level interpersonal perceptions (perceived team-member exchange and perceived coworker support) as key contextual factors related to OCB-H continuity, shifting focus from the well-researched leader-member exchange (LMX) to horizontal team-member exchange (TMX)—an understudied but vital dimension of workplace social exchange (Banks et al., 2014; Tano et al., 2023). This focus addresses the lack of research on how equal, peer-to-peer relationships shape the persistence of prosocial behavior in teams. Third, it provides practical insights for organizational management by unpacking how team relationship perceptions may be internalized by employees to relate to sustained helping behavior. Unlike prior research that guides managers to focus on leader-subordinate relationships, this study highlights the importance of nurturing positive peer-to-peer dynamics, offering actionable guidelines for optimizing team cohesion and sustaining voluntary prosocial behavior.

In summary, this study constructs a dynamic mediated-moderation model to examine the persistence of employee helping behaviors within an organization as well as the external influences. Based on social exchange theory, we argue that good team-member exchange relationships may foster a sense of coworker support among employees, which in turn may strengthen the persistence of OCB-H—illustrating how external team relationship perceptions may be internalized to relate to sustained prosocial behavior. This paper is structured as follows: Section 2 presents the theoretical background and research hypotheses. Section 3 describes the sample characteristics, presents the measurement method, and reports the data analysis method. Section 4 reports the results of the data analysis, and Section 5 discusses the results of the present study and points out the limitations of the study and suggestions for future research.

## Theoretical foundation and hypothesis development

### Continuity of organizational citizenship helping behavior

Existing models focus on static predictors, not dynamic continuity. Stable between-person variance, such as positive versus negative personality tendencies, internal and external attributional styles can make a difference to an individual’s attitude and performance at work. However, these studies have been conducted from a static perspective, ignoring the differences in performance of the same individuals at different points in time as a result of the dynamics of time. George and Jones (2000) note that ‘time can completely change the way theoretical ideas and their relationships are conceptualized, and thus the propositions that are derived from theories’. As can be seen, exploring the changes presented by variables over time is receiving increasing research attention, and time is becoming an important influence variable in organizational relationships (Matta et al., 2020; Ju et al., 2019; Qin et al., 2018a; Qin et al., 2018b). Therefore, introducing the perspective of intra-individual variance (within-person variance) and the perspective of time to interpret the changes in behavior is necessary (Van den Broeck et al., 2021). For the change in organizational citizenship behavior, the typical internal interpretation perspective is the emotional mechanism. Previous studies show the most important cause of the change in organizational citizenship behavior of the same individual in different occasions is individual’s emotional state (Chatzopoulou et al., 2022; Dalal et al., 2009). This paper distinguishes itself by offering a multifaceted theoretical lens, analyzing the persistence of OCB-H through the reciprocity perspective of social exchange theory, while also integrating insights from social cognitive theory and conservation of resources theory to provide a more comprehensive understanding.

Social exchange theory (Blau, 1964) points out that social exchange behavior is based on interpersonal interaction, and exchange behavior can be defined as ‘behavior that occurs when the other party responds reciprocally and stops when they do not’. This definition suggests that exchange behavior exists in interpersonal interactions and that when exchange behavior receives timely and positive feedback, the behavior has a high tendency to be sustained. In organizational studies, mutual help among employees, such as helping a colleague who is on leave to complete a work task or assisting a supervisor in completing an assigned task, are typical reciprocal exchange behaviors (Zeb et al., 2023; Schuh et al., 2018). The law of reciprocity can be viewed as the mechanism by which social exchange occurs and terminates, and if one party does not reciprocate and breaks the norm of reciprocity, continuing the exchange behavior is difficult. Reciprocity is a powerful source of motivation, and it has significant effect on individual job satisfaction and organizational commitment, which are important antecedents of organizational citizenship behavior (Pham et al., 2023).

From the perspective of social cognitive theory (Bandura, 1993), individuals’ behavior is influenced by their cognitive processes, including their perceptions, expectations, and self-efficacy beliefs. In the context of OCB-H, social cognitive theory suggests that employees who have previously engaged in helping behaviors are likely to develop positive self-perceptions and expectations about the outcomes of their actions. If an employee perceives that their helping behavior has been positively received by colleagues and supervisors, they are likely to develop a sense of self-efficacy in their ability to contribute positively to the organization (Woreta et al., 2025), thereby increasing their likelihood of engaging in similar behaviors in the future. These cognitive processes, in turn, reinforce their motivation to continue engaging in helping behaviors.

Furthermore, conservation of resources theory (Hobfoll, 1989) provides additional insights into the persistence of OCB-H. According to this theory, individuals accumulate resources over time through their interactions and experiences within the organization. These resources can include tangible assets such as knowledge and skills, as well as intangible assets such as social capital and reputation (Hobfoll et al., 2018). When employees engage in helping behaviors, they not only contribute to the well-being of their colleagues but also accumulate resources in the form of social capital and positive reputation. These accumulated resources, in turn, enhance their ability to engage in future helping behaviors by providing them with the necessary support, information, and influence within the organization. Therefore, more helping behaviors an employee engages in, more resources they accumulate, and the higher their likelihood of continuing to engage in such behaviors in the future.

When employees demonstrate positive helping behaviors to their colleagues, under the incentive of reciprocity, individuals may experience higher job satisfaction and organizational commitment with other positive responses, which may stimulate their next phase of helping behaviors. Moreover, organizational citizenship and helping behaviors tend to continue with the norm of reciprocity. Drawing on social cognitive theory and conservation of resources theory, it can be argued that the persistence of OCB-H is not only driven by the immediate rewards and reciprocity but also by the long-term cognitive and resource-based benefits that employees derive from their helping actions. Therefore, this paper proposes the first hypothesis based on the reciprocity perspective:

*Hypothesis 1: Organizational citizenship helping behavior in Phase-1 is positively associated with that in Phase-2,* i.e.*, the more organizational citizenship helping behavior exhibited in Phase-1, the higher the likelihood of exhibiting helping behaviors in Phase-2.*

### Moderating effects of perceived team-member exchange

Team members often share team goals and tasks and are jointly influenced by the organization’s culture and resources (Banks et al., 2014; Liden et al., 2014). Seers (1989) first developed the concept of team-member exchange to describe the quality of relationships that individual members perceive from colleagues around them. Team-member exchange reflects the extent to which team members share ideas and feedback with each other and recognize each other’s competence and role as team members. Respect for each other’s ideas and feedback, recognition of others, and assisting each other in daily work all reflect good member exchange (Banks et al., 2014). Team member exchange relationships can also be seen as the aggregation of horizontal exchanges between team members (Tano et al., 2023; Farmer et al., 2015). In addition, Martínez-Tur et al. (2020) suggested that team member exchange relationships can measure the degree of reciprocity among team members. Thus, with the process of social exchange, team members will follow the principle of reciprocity and attempt to maintain the balance of social exchange between each other.

High-quality team member exchange relationships also may significantly increase employees’ motivation to engage in organizational citizenship behaviors (Reader et al., 2017; Hu and Liden, 2013). According to Blau’s definition of social exchange, whether or not an employee exhibits sustained supportive behavior depends on his or her own measure of ‘whether or not he or she gets something in return’. When individuals perceive high quality of member exchange, members communicate more efficiently and are able to make other suggestions of each other’s work. Recognition of work and good teamwork atmosphere improve the quality of communication between team members. With good cooperative atmosphere, employees perceive that their colleagues give them additional positive feedback, which may potentially enhance organizational citizenship behaviors. Good exchange relationships optimize employees’ behavioral performance. By contrast, when employees perceive team member exchange relationships at a lower level, they are more likely to feel work pressure. Additionally, their willingness to leave the job is significantly higher (Harden et al., 2021; Carter et al., 2013). Poor team member exchange relationships also indicate that individuals do not receive positive feedback from other team members. Moreover, the law of reciprocity between coworkers no longer exists, which reduces the likelihood of subsequent organizational citizenship supportive behaviors.

Both the above theoretical perspectives and empirical studies illustrate that perceived good or bad team member exchange relationships bring different interpersonal experiences to individuals, and that good team member exchange perceptions imply the existence of benign reciprocal exchanges within the team. Thus, mutual supportive behaviors may be more likely to continue. Based on the above analysis, this paper proposes the following hypothesis:

*Hypothesis 2: Perceived team-member exchange moderates the positive association between Phase-1 and Phase-2 organizational citizenship helping behavior,* i.e.*, better perceived team-member exchange, stronger positive linksbetween the two phases.*

### Moderating and mediating role of perceived coworker support

Perceived coworker support stems from the concept of organizational sense of support, which reflects the mutual measurement of exchange relationships between employees. The sense of organizational support was first proposed by Eisenberger, emphasizing the commitment of the organization to its employees. Perceived coworker support is an internalized psychological perception that employees form based on the external team relational climate—reflecting the extent to which employees subjectively feel that colleagues value their contributions and care about their well-being (Halbesleben and Wheeler, 2015). According to social exchange theory, the principle of reciprocity plays a crucial role in social interactions. When one party in a non-explicitly obligatory relationship gives the other party more than the usual facilities or rewards, a common feeling is established between them, which is further strengthened in the process of reciprocity between the two pairs. High quality team member exchange relationship means shared emotions, representing mutual trust and respect. Perceived team member exchange is one’s overall perception of the team’s interpersonal relationships, which is a relatively stable relational environment formed by long-term interaction among team members (Uhl-Bien et al., 2022; Banks et al., 2014). Shore et al. (2009) further stated that good relationships between coworkers are demonstrated by positive responses and reciprocal interpersonal processes, as well as the ability to exchange resources and provide positive feedback during team member interactions. High quality team member exchange relationship has positively oriented effect on employees’ processing of information, i.e., team members will be willing to believe in the team’s ability to work together and in the achievability of goals (Gong et al., 2013).

The perceived good or bad team member exchange relationship will directly relate tot the cognitive experience of individuals in the team. Employees need to continuously accumulate and verify information about team member exchange through daily work interactions before they can form a stable and consistent perception of coworker support. When the team members’ exchange relationship is good, further exchanges happen between coworkers with adequate information transfer, a solid and pleasant common emotion can be established. Additionally, everyone will respect and trust each other, individuals can perceive extra support and encouragement from their coworkers. Conversely, when the members’ exchange relationship is poor, reciprocity is no longer reciprocal, both parties no longer exchange opinions and resources equally. Meanwhile, the common emotional foundation is broken, and the perceived mutual trust and support is significantly reduced. Based on this assumption, this paper proposes the following hypothesis:

*Hypothesis 3: Perceived team-member exchange is positively associated with perceived coworker support,* i.e.*, perceived coworker support tends to be higher when perceived team-member exchange is better.*

Employees’ perceived organizational support is closely related to job involvement, organizational commitment, performance, and organizational citizenship behavior (Chernyak-Hai et al., 2024; Halbesleben and Wheeler, 2015). The relationship between employees’ perceived support and behavioral performance is also based on social exchange theory and the principle of reciprocity. The formal motivation generated by the feeling of support creates a ‘sense of indebtedness’ for the individual, which in turn creates a corresponding obligation to give back to the organization. Owing to the autonomous nature of organizational citizenship, it can be a way of fulfilling these obligations and further reinforces the intrinsic value of the exchange relationship (Kim et al., 2020). Once team member perceives the support from co-workers, the high likelihood he/she will pay back to them, which can enhance the mutual support behavior among co-workers (Chernyak-Hai et al., 2024). Colleagues express mutual concern, respect, and solidarity, they also perceive each other’s support for themselves, thereby creating high value and durability to the exchange relationship. When individuals perceive that colleagues have a high degree of support for themselves, they are willing to show friendly attitudes and behaviors toward their colleagues and are willing to perform their own good performance to fulfill the reciprocal obligations in the process of working together. By contrast, when individuals perceive a reduced sense of support, the principle of reciprocity is broken. Employees are no longer willing to give unilaterally in their emotions, and the helping behaviors will be reduced or even disappear. Based on this assumption, this paper proposes the following hypothesis:

*Hypothesis 4: Perceived coworker support moderates the positive association between Phase-1 and Phase-2 organizational citizenship helping behavior,* i.e.*, higher perceived coworker support is associated with a stronger positive link between the two phases.*

According to the above analysis, the quality of interpersonal relationships in a team is key to an employee’s willingness to consistently be the “good people” in the organization—to consistently provide help to colleagues. Emotional support from team members increases employees’ self-efficacy and generates positive emotions in the workplace (Zhu et al., 2019). When employees feel the support of others in the organization, they work harder in return, become more productive, and develop more identification with the organization. A high level of identification with the organization creates a secure and supportive network of relationships, which also reduces the emotional anxiety and stress associated with work tasks, thus improving performance (Boies and Howell, 2006). The positive and sustained effects of high-quality exchange relationships between co-workers on OCB-H are somewhat mediated by co-workers’ emotional support and one’s own sense of identification with the organization. Therefore, this study suggests that the moderating effect of perceived team member exchange relationships on the persistence of helping behaviors is transmitted through perceived coworker support, as a mediating transmission mechanism. In other words, perceived coworker support transmits the moderating effect of the original moderator variable, namely perceived team coworker exchange relationships.

The above inferences and the theoretical derivation of Hypotheses 2, 3, and 4 are combined. According to the definition of the mediated moderating effect, the mediated moderation begins with a moderating relationship, and this moderating effect constitutes the mediated moderating effect when it can be transmitted through the mediating variable (Edwards and Lambert, 2007). This study proposes such a mechanism of action as follows:

1. A variable (perceived team-member exchange) moderates the association between the independent variable (Phase-1 OCB-H) and the dependent variable (Phase-2 OCB-H), i.e., Hypothesis 2.
2. The moderating variable (perceived team-member exchange) relates to the mediator variable (perceived coworker support), i.e., Hypothesis 3.
3. The mediating variable (perceived coworker support) moderates the association between the independent variable (Phase-1 OCB-H) and the dependent variable (Phase-2 OCB-H), i.e., Hypothesis 4.

Perceived coworker support plays a bridging role throughout the continuation of helping behaviors. When employees form a stable perception of coworker support, this internalized psychological perception will generate a sense of reciprocity obligation (Kim et al., 2020), which directly relates to sustained helping behavior at the same time point. This integrated mediated-moderation framework represents a new theoretical perspective for understanding the dynamic continuity of OCB-H, rather than merely testing direct or indirect effects. Based on the above assumption, this paper hypothesizes the following:

*Hypothesis 5: The moderating effect of perceived team-member exchange on the link between two-phased organizational citizenship helping behavior is transmitted through employee-perceived coworker support. That is, perceived team-member exchange strengthens the link between two-phased organizational citizenship helping behavior, by enhancing perceived coworker support.*

**Figure 1:**
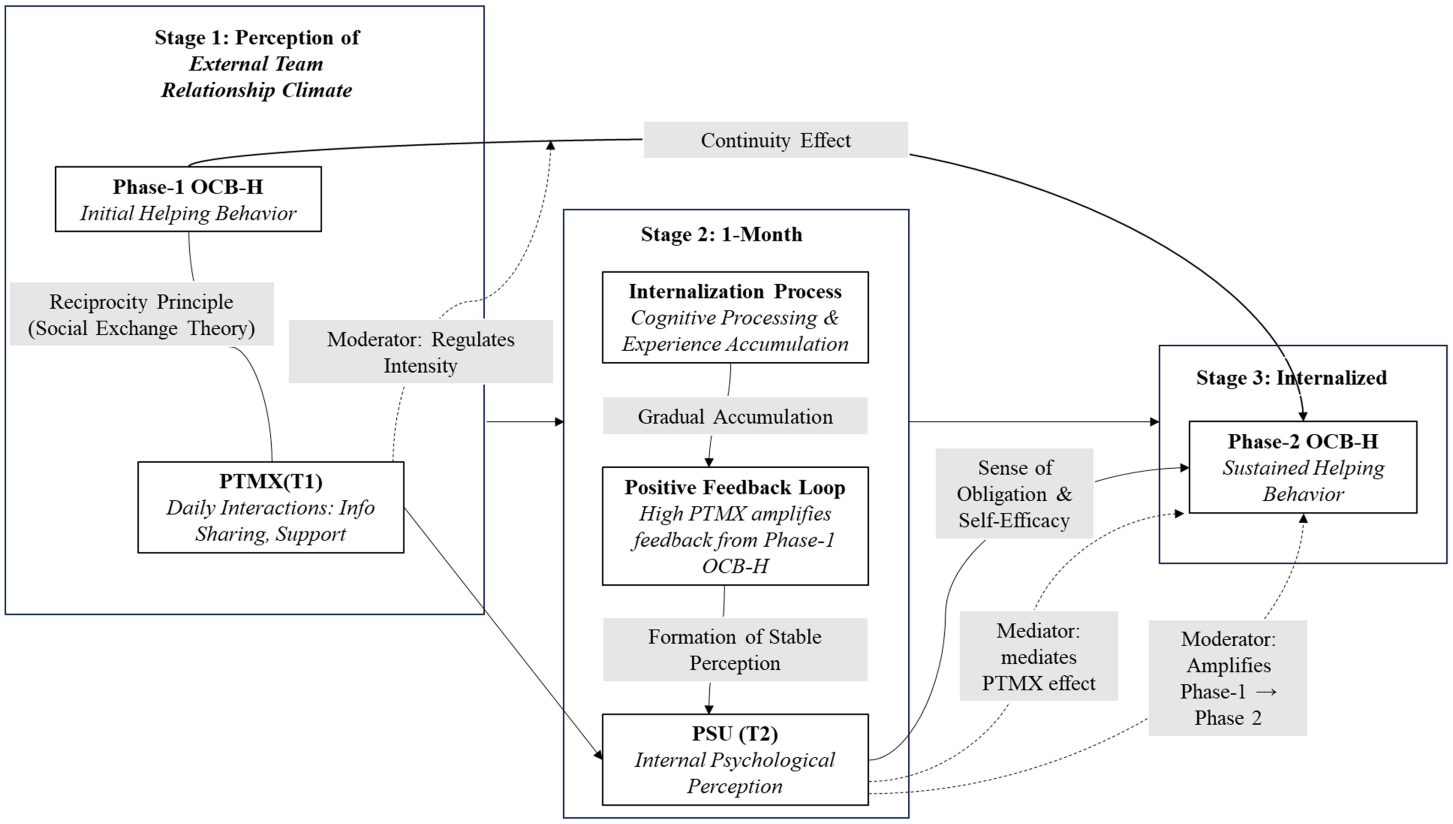
Theoretical process. T1 = Phase 1, T2 = Phase 2. OCBH = Organizational citizenship helping behavior, PTMX = perceived team-member exchange, PSU = perceived coworker support; → direct effect; 
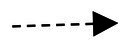
 moderate/mediate effect.

Figure 1 shows the dynamic psychological internalization process of this study and marks the temporal nodes (Time 1 and Time 2) and causal logic chain of each construct.

**Figure 2:**
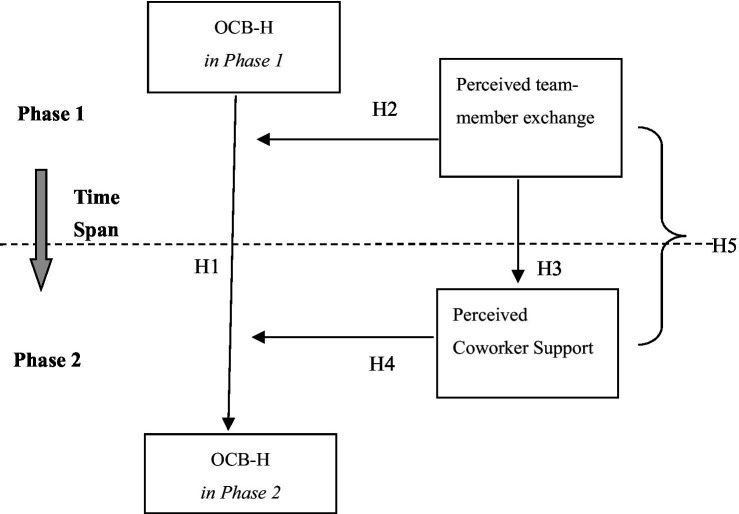
Conceptual model. OCB-H refers to “Organizational Citizenship Helping Behavior.”

In summary, this paper proposes a dynamic mixed model that spans time points and includes the mediated moderation effect as shown in Figure 2.

## Method

### Participants and procedure

The sample of this study originates from private enterprises mostly from Jiangsu Province in China, which covers several industries, i.e., manufacturing, high-tech, and logistics industries. We processed the data by encoding each employee. Before filling out the questionnaire, every participant in the survey signed an informed consent form. To reduce potential homogeneous variance, this paper utilizes a longitudinal research design with a phased approach to data collection. We distributed the first batch of questionnaires for measuring variables such as OCB-H, perceived team-member exchange to 1,000 employees at time point 1, and recovered 782 valid questionnaires with an effective rate of 78.2%.

Following prior research (Dalal et al., 2009), we adopted a 1-month interval between measurement waves to balance temporal precision and participant retention. Short intervals minimize recall bias and confounding events while still allowing for causal inferences in longitudinal designs. The second batch of questionnaires was distributed to the 782 employees to measure the variables of OCB-H and perceived coworker support at time point 2 one month later. Six hundred and twenty valid questionnaires were recovered with an effective rate of 79.3%.

Among the above valid questionnaires, a total of 394 are male employees (63.6%) and 226 are female (36.4%). Among them, 63.4% are below 30 years old, 33.7% are 30 to 40, and 2.9% are above 40. In terms of educational level, 22.5% have a vocational or lower education, 25.0% have a college education, 43.9% have a bachelor’s degree, and 8.6% have a graduate degree or higher.

### Measures

The specific measures are described below. We provide the coefficients of Cronbach’s alpha, composite reliability (CR) and average variance extracted (AVE) for each measure. All survey responses were made on a 5-point scale (1 = strongly disagree, 5 = strongly agree).

#### Organizational citizenship helping behavior

We captured OCB-H with 5 items developed by Coyle-Shapiro (2002). For example, ‘I give my time to help coworkers who have a heavy workload’ and ‘I used to help coworkers on leave to finish their work’. The internal consistency coefficient is 0.725 for Phase-1 and 0.750 for Phase-2, CR is 0.82 and 0.93, AVE is 0.48 and 0.58, respectively. Notably, the AVE of Phase-1 OCB-H was 0.48, which is marginally below the conventional threshold of 0.50. This is common and acceptable in longitudinal and dynamic research focusing on within-person changes in early-stage behavioral measurement. Given that the composite reliability (CR = 0.82) was satisfactory and the scale demonstrated sound temporal stability and predictive validity, the measurement remained appropriate for testing the proposed model.

#### Perceived team-member exchange

This study used 10 items to measure employees’ ratings of their perceived team-member exchange (Seers et al., 1995). Example items were‘I often advise other team members on optimizing work methods’, ‘They always let me know when my work makes other team members’ work easier (or more difficult)’, ‘In order to make other team members’ work easier, they always let me know’, ‘I have the flexibility to adjust my job duties in order to make other team members’ jobs easier’, and ‘Other team members understand my problems and needs’. The internal consistency coefficient is 0.884, CR is 0.96 and AVE is 0.71.

#### Perceived coworker support

We used a 10-item scale developed by Ducharme and Martin to measure employees’ perceived coworker support (Ducharme and Martin, 2000). Example items were ‘My coworkers genuinely care about me’, ‘I feel appreciated by my coworkers’, ‘My coworkers help me in the process of completing my work’, and ‘My coworkers assist me when faced with uncommon work challenges’. The internal consistency coefficient is 0.938, CR is 0.85 and AVE is 0.54.

#### Control variables

In this paper, demographic variables like gender (male = 1, female = 0), age (20 years old and below = 1, 21–25 years old = 2, 26–30 years old = 3, 31–35 years old = 4, 36–40 years old = 5, 41–45 years old = 6, and 46 years old and above = 7), education (middle school and below = 1, junior high school or technical secondary school = 2, senior high school = 3, junior college = 4, bachelor’s degree = 5, and master’s degree and above = 6) were used as control variables. In addition, job tenure (years in the position), monthly income (less than RMB3,000 = 1, 3,001–5,000 = 2; 5,001–7,000 = 3; 7,001–9,000 = 4; 9,001–11,000 = 5; 11,001–13,000 = 6; 13,001–15,000 = 7; 15,001 and above = 8), and team size (i.e., number of team members) were also used as control variable (Ozer et al., 2014).

These variables were controlled for their potential confounding effects: age is linked to work experience and social networks that shape perceived team-member exchange, perceived coworker support and OCB-H propensity (Ng and Feldman, 2012); income reflects economic status, affecting cognitive resources for prosocial behavior and perceived organizational status (Deckop et al., 1999); education is associated with cognitive ability and workplace relationship expectations that influence perceived team-member exchange, perceived coworker support and cooperative behaviors (Arvey et al., 1992); team size structures team dynamics, communication and relational cohesion, impacting interpersonal perceptions and helping behavior (Hackman, 2002); job tenure represents organizational embeddedness, shaping established colleague relationships, team norm knowledge and sense of belonging (Morrison, 2002). Controlling for these factors isolates the unique effects of the focal variables in the study.

### Validity of the data structure and common method bias test

We used LISREL 8.80 to conduct a validated factor analysis (CFA) to test the discriminant validity among the variables. Using the 4-factor model (all variables independently separated) as the baseline, three competing models were constructed as follows. A 3-factor model (employee-perceived team-member exchange and coworker support attributed to one latent variable, plus Phase-1 OCB-H, and Phase-2 OCB-H), and a 2-factor model (Phase-1 OCB-H, employee-perceived team-member exchange, and coworker support attributed to one latent variable, plus Phase-2 OCB-H), and a 1-factor model (the four factors were perfectly correlated with correlation coefficients set to 1).

To further address potential common method bias concerns, we also conducted a Harman single-factor test using CFA. In this test, we forced all measured variables to load on a single latent factor. The results showed a poor fit for this 1-factor model, with fit indices (such as χ^2^/df, RMSEA, CFI, etc.) significantly worse than those of our baseline 4-factor model. This indicates that a single factor does not adequately explain the variance in our data, providing evidence against the presence of severe common method bias. Thus, the Harman single-factor test results support the discriminant validity of our constructs and the robustness of our findings. Discriminant validity was comprehensively evaluated through confirmatory factor analysis and model comparison. The hypothesized four-factor model yielded significantly better fit indices than all alternative models, supporting the distinctiveness of each construct. In addition, all composite reliability values exceeded the corresponding AVE values, providing further evidence of acceptable discriminant validity.

The results showed (Table 1) that the fit indexes in the 4-factor model are significantly better than the other three models (χ^2^/df = 4.001, RMSEA = 0.071, CFI = 0.955, NFI = 0.941, NNFI = 0.945), which initially demonstrated that the four latent variables were different constructs.

**Table 1:** Validation factor analysis.

Model	χ^2^	Df	χ^2^/df	RMSEA	CFI	NFI	NNFI
1-factor model	2,848.082	364	7.824***	0.106	0.897	0.884	0.877
2-factor model	2,422.650	362	6.692***	0.098	0.912	0.899	0.894
3-factor model	2,102.151	359	5.856***	0.093	0.922	0.909	0.906
4-factor model	1,420.184	355	4.001***	0.071	0.955	0.941	0.945

*N* = 620; ***p < 0.001.

## Results

### Descriptive statistics and correlation analysis

Table 2 shows the means, standard deviations, Pearson’s correlation coefficients, and significance levels of all variables. The two phases of OCB-H are significantly and positively correlated (*γ* = 0.334, *p* < 0.01), and that employees’ perceived team-member exchange is significantly and positively correlated with coworker support (γ = 0.173, *p* < 0.01). The significant correlations between these main variables provide a foundation for subsequent analyses.

**Table 2:** Means, standard deviations, Pearson’s correlation coefficients, and significance levels.

Variables	Mean	SD	1	2	3	4	5	6	7	8	9
Gender (T1)	0.630	0.482									
Age (T1)	3.330	1.000	0.071								
Edu (T1)	4.220	1.227	−0.009	0.177**							
Tenure (T1)	5.480	3.882	0.009	0.525**	−0.207**						
Income (T1)	3.160	1.516	0.054	0.075	−0.140**	0.094*					
TeSize (T1)	3.760	1.051	−0.018	−0.019	−0.015	−0.014	−0.118**				
OCBH (T1)	4.327	0.604	−0.010	0.053	−0.033	0.129**	−0.017	0.000			
PTMX (T1)	4.102	0.583	0.072	0.142**	0.017	0.104*	−0.008	−0.058	0.597**		
PSU (T2)	3.958	0.736	−0.067	0.039	−0.029	0.070	0.037	0.022	0.168**	0.173**	
OCBH (T2)	4.194	0.547	0.048	0.103*	0.016	0.023	−0.037	0.051	0.334**	0.294**	0.283**

*N* = 620; **p < 0.01, *p < 0.05; T1 = Phase 1, T2 = Phase 2, OCBH = Organizational citizenship helping behavior, PTMX = Perceived team-member exchange, PSU = Perceived coworker support; Edu = Education background, TeSize = Team size.

A variance inflation factor (VIF) analysis was further conducted for all regression variables (including controls, independent, mediator and moderator) to assess multicollinearity risks, with results confirming no such issues: the average VIF of all variables is 1.32, all individual VIF values (the highest being 1.76 for organizational tenure) are well below the universal threshold of 10 (Hair et al., 2019). The VIF values of key focal variables are 1.21 (PTMX), 1.35 (PSU) and 1.18 (OCB-H T1), respectively. These results verify no high correlation among model variables, ensuring the validity of regression coefficients and statistical inferences.

### Hypothesis testing

In this paper, the sample data were first analyzed by using the SPSS21.0 software to conduct hierarchical regression analysis. To meet the requirements of hypothesis testing, a total of six models were set up. Table 3 shows the specific results. Among them, the explanatory variable of Model 1 to 4 is Phase-2 OCB-H, and the explanatory variable of Models 5 and 6 is perceived coworker support.

**Table 3:** Hierarchical regression results.

Variables	Phase-2 organizational citizenship helping behavior				Perceived coworker support	
	Model 1 (H1)	Model 2 (H2)	Model 3 (H4)	Model 4 (H5)	Model 5 (H3)	Model 6 (H5)
Gender	−0.064(−1.345)	−0.083 + (−1.701)	−0.044(−0.931)	−0.066(−1.372)	−0.128*(−1.972)	−0.115 + (−1.780)
Age	0.088**(2.920)	0.071*(2.348)	0.082**(2.782)	0.069*(2.302)	0.035(0.869)	0.032(0.797)
Edu	0.000(−0.110)	0.006(0.279)	0.003(0.077)	0.006(0.318)	−0.019(−0.695)	−0.011(−0.402)
Tenure	−0.014 + (−1.930)	−0.013 + (−1.783)	−0.015*(−2.100)	−0.014 + (−1.937)	0.001(0.062)	0.001(0.056)
Income	−0.012(−0.774)	−0.009(−0.585)	−0.017(−1.088)	−0.013(−0.862)	−0.001(−0.031)	0.002(0.104)
TeSize	0.052*(2.308)	0.060**(2.641)	0.039 + (0.078)	0.047*(2.077)	0.078*(2.578)	0.077*(2.563)
OCBH(T1)	0.256***(6.816)	0.208***(4.300)	0.201***(5.201)	0.167**(3.398)		0.136*(2.112)
PTMX		0.144**(2.809)		0.119*(2.341)	0.264***(4.844)	0.193**(2.833)
OCBH(T1)*PSU			0.140*(2.063)	0.100(1.420)		
OCBH(T1)*PTMX		0.198*(2.538)		0.130(1.631)		0.257*(2.476)
PSU			0.156***(4.525)	0.133***(3.749)		
*R* ^2^	0.120	0.152	0.171	0.185	0.072	0.088
Adjusted *R*^2^	0.107	0.135	0.155	0.165	0.058	0.069
△*R*^2^	0.087	0.012	0.008	0.004	0.047	0.012
*F*	9.205***	9.041***	10.645***	9.243***	5.074***	4.833***

*N* = 620; **p < 0.01, *p < 0.05, +*p* < 0.1; T1 = Phase 1, T2 = Phase 2, OCBH = Organizational citizenship helping behavior, PTMX = Perceived team-member exchange, PSU = Perceived coworker support; Edu = Education background, TeSize = Team size. The values in the brackets are the *t*-values.

As shown in Table 3, Model 1 tests the main effect of Phase-1 OCB-H on Phase-2. The results show the main effect is significant with a medium effect size after controlling for the relevant variables (*β* = 0.256, *p* < 0.001, △R^2^ = 0.087), and Hypothesis 1 holds. Model 2 tested the moderating-effect of employees’ perceived team-member exchange on the relationship between the two phases of OCB-H, adding moderating variables as well as an interaction term to Model 1. The moderating effect of perceived team-member exchange on the two-phase helping behavior is significant with a small effect size (β = 0.198, *p* < 0.05, △R^2^ = 0.012), which is consistent with the common intensity characteristics of team contextual variables’ moderating effects on employees’ voluntary behaviors in organizational behavior research, led to the establishment of Hypothesis 2. Model 5 showed that after controlling for relevant variables, the direct effect of perceived team-member exchange on coworker support is significant with a medium effect size (*β* = 0.264, *p* < 0.001, △R^2^ = 0.047), and Hypothesis 3 was established. Model 3 shows that the moderating effect of perceived coworker support on the two-phase helping behavior is significant with a small effect size (*β* = 0.140, *p* < 0.05, △*R*^2^ = 0.008) through the regression coefficient of the interaction term (Phase-1 OCB-H × perceived coworker support) on Phase-2 OCB-H, suggesting the existence of a moderating effect, thus supporting Hypothesis 4.

Hypothesis 5 proposes a moderated-mediating hypothesis. Testing the hypothesis requires meeting the following conditions: (i) the regression coefficient of the interaction term (Phase-1 OCB-H × perceived team-member exchange) on the dependent variable (Phase-2 OCB-H) is significant; (ii) the regression coefficient of the interaction term (Phase-1 OCB-H × perceived team-member exchange) on the mediator variable (perceived coworker support) is significant; and (iii) the mediator variable (perceived coworker support) has a significant regression coefficient on the dependent variable (Phase-2 OCB-H), and the coefficient of the interaction term (Phase-1 OCB-H × perceived team-member exchange) becomes insignificant or decreases. If the coefficient becomes insignificant, it indicates that perceived coworker support plays a fully mediating role; if the coefficient decreases, it indicates a partially mediating role. Model 6 shows that the interaction term has a significant regression coefficient on perceived coworker support (*β* = 0.257, *p* < 0.05). In Model 4, the regression coefficient of perceived coworker support is significantly associated with Phase-2 OCB-H (*β* = 0.133, *p* < 0.001), and the coefficient of the interaction term became insignificant (*β* = 0.100, *t* = 1.420), suggesting that perceived coworker support plays a fully mediating role in the moderating effect of perceived team-member exchange on the link between the two-phased OCB-H, thus supporting Hypothesis 5.

Owing to the possible Type II Error associated with the causal step method, this paper further utilizes the process plug in SPSS 21.0 to conduct 5,000 Bootstrap sampling of the full sample, to further examine the reasonableness of the mediated regulation model in this paper (Edwards and Lambert, 2007; Ye and Wen, 2013). The effect size of the mediated moderation effect is 0.047 with a standard error of 0.015, and the 95% bias-corrected confidence interval does not contain 0 (LLCI = 0.023, ULCI = 0.087), which is consistent with the statistical significance of the mediated moderation effect. Moreover, the narrow interval range indicates that the estimation result of the mediated moderation effect size in this study has high stability and reliability.

**Figure 3:**
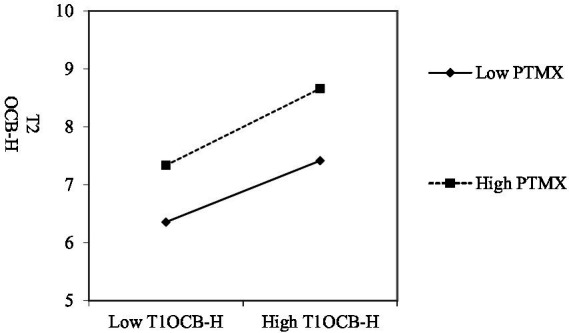
Phase-1 OCBH and PTMX with phase-2 OCBH (H2 supported).

**Figure 4:**
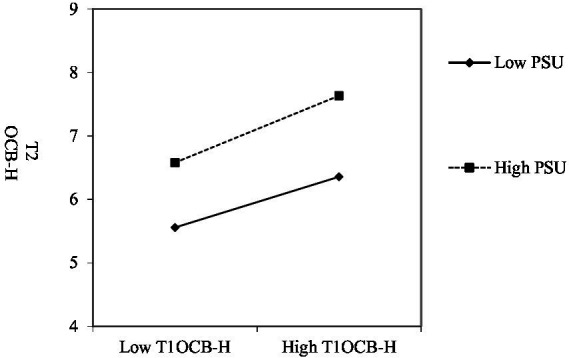
Phase-1 OCBH and PSU with phase-2 OCBH (H4 supported).

In addition, this paper refers to the method provided by Aiken et al. (1991) to plot the interaction effects to illustrate more clearly the moderating effect of perceived team-member exchange and coworker support on the persistence of organizational citizenship helping behaviors. As can be seen from Figure 3, when employees’ perceived team-member exchange has higher quality, the positive link of Phase-1 OCB-H to Phase-2 is significantly stronger, supporting Hypothesis 2. In Figure 4, when employees’ perceived coworker support is stronger, the positive link of Phase-1 OCB-H to Phase-2 is significantly stronger, supporting Hypothesis 4. This finding suggests that when employees perceive a high quality of team member exchange relationship, they are more willing to give consistently and provide helping behaviors to others. Similarly, if individuals perceived extra support from their colleagues, it also promotes their consistent positive behaviors.

## General discussion

In the discussion of the continuity mechanism of organizational citizenship helping behavior, we directly reference the empirical results from Phase-1 and Phase-2 data. The results consistently supported the proposed model. First, the direct effect showed that Phase-1 OCB-H was positively associated with Phase-2 OCB-H (*β* = 0.256, *p* < 0.001), supporting behavioral continuity. This finding aligns with social exchange theory, which posits that reciprocal relationships foster sustained behavioral patterns (Blau, 1964). Second, two moderating effects showed that PTMX and PSU strengthened the temporal continuity of helping. The moderating role of perceived team-member exchange (PTMX) is evident in its enhancement of OCB-H continuity (β = 0.198, *p* < 0.05), indicating that high-quality interpersonal relationships within teams may act as a significant facilitator for the continuation of altruistic behaviors. Third, the mediated moderation effect revealed that PTMX enhanced behavioral continuity by increasing PSU, which could potentially translate the contextual influence into sustained motivational factors.

However, our study uniquely notes that some beta coefficients in this study have small absolute values but are statistically significant. On the one hand, this is due to the high statistical power brought by the 620 valid samples; on the other hand, it is related to the multi-factor influencing attribute of organizational citizenship behavior. As a spontaneous altruistic behavior of employees, its continuity cannot be strongly explained by a single contextual variable or interaction term. Significant results with small effect sizes are more in line with the actual influencing laws of employee behavior in reality, and such small effect sizes have universal practical reference value in relevant research on team relationships and organizational citizenship behavior (Banks et al., 2014).

### Theoretical implications

The core theoretical novelty of this study lies in moving beyond static, one-time explanations of OCB-H to build a dynamic, time-sensitive model of behavioral continuity. By constructing a dynamic mediated moderation model, we reveal how external interpersonal relationships (PTMX) may be internalized into perceived support (PSU), which in turn reinforces helping behaviors through self-efficacy and resource accumulation mechanisms (Hobfoll, 1989; Bandura, 1993). This process-oriented explanation fills a gap in prior research, which often treated OCB persistence as a static outcome rather than a dynamic interplay of cognitive, emotional, and relational factors. Our theoretical contributions can be summarized clearly as follows:

First, this study re-conceptualizes OCB-H as a temporally sustained behavior rather than a static discrete act. While prior studies (e.g., Mo et al., 2023) have emphasized static antecedents of organizational citizenship behavior, we adopt a dynamic perspective by examining changes in helping behavior over time. The empirical research in this paper shows that OCB-H at the first time point has a significant positive association with that at the second time point, demonstrating the continuity of individual behaviors based on temporal changes. This finding also supports the research on the dynamics of organizational citizenship behaviors interpreted from the internal perspective, suggesting that good team member exchange relationship can be transformed into identification with oneself through cognition, which in turn promotes the performance of altruistic behaviors. In contrast to the external-difference research perspective, the internal-difference perspective provides a better interpretation of the process mechanism of behavior change. Such a dynamic perspective is inspirational for the theoretical construction and research methods innovation in future studies.

Second, this study establishes a novel peer-centered relational pathway that complements dominant leader-centric (LMX) models. Our findings contrast with those of Zhang et al. (2024), who found limited impact of peer relationships on OCB in hierarchical settings, by demonstrating that perceived coworker support fully mediates the link between perceived team-member exchange and OCB-H persistence in flat team structures. Empirical findings show that ‘good people’ in the workplace tend to maintain being good. However, team contextual boundaries also exist. When employees show positive altruistic behaviors at the first time point, better team member exchange relationships will promote their continued role as ‘good people’ at the next time point. In addition, good team member exchange relationships will enhance their ‘inertia’ in organizational citizenship helping behaviors through their interpersonal relationships. When employees show positive altruistic behavior in phase 1, that will promote them to continue playing the role of ‘good’, and enhance the ‘inertia’ of their organization’s civic supportive behavior. Good team member exchange relationship will only promote the further continuation of the interpersonal supportive behavior, through one’s perception and internalization. In other words, a team with the good communication environment and resource sharing will make enthusiastic colleagues be willing to maintain enthusiasm, help team members, and improve the efficiency and performance of the organization. Therefore, contextual boundaries of team members’ continuous altruistic behaviors exist under the perspective of interpersonal relationships, and friendly interpersonal relationships are the foundation for ‘good people’ to continue being good.

Third, this paper dynamically provides supportive evidence for the transformation process of employees from external relationship perception to self-identity. This is not merely moderation and mediation, it is a two-stage internalization process that explains how relational perceptions become sustained motivation. Unlike previous research that focused solely on individual-level factors (e.g., proactive personality; Yang et al., 2011), our model integrates team-level variables (perceived team-member exchange and perceived coworker support), revealing their cascading effects on behavioral continuity. This study combines the perspectives of individual internal differences and behavioral dynamics, to reveal the dynamic integration process in which external perceptions are internalized and further manifested as good behaviors in team collaboration scenarios. According to the results of the empirical tests, to magnify the positive impact of the exchange relationships among team members depends on whether such exchange relationships can be internalized into the degree of support that employees perceive from their colleagues. Which is more, we provide supportive evidence that perceived coworker support acts as a full mediator—a finding inconsistent with Kumar’s partial mediation model (Kumar et al., 2021). although the moderating effect of PSU (*β* = 0.140, *p* < 0.05) is a small effect, it reveals the key mediating path of ‘individual internalization of team relationship perception into behavioral motivation’, which is the core link of the theoretical model in this study and has important theoretical incremental significance. These discrepancies underscore the importance of contextual factors in shaping OCB outcomes. Once employees internalize the harmonious interpersonal relationships through their own perceptions and translate them into a sense of support from coworkers, they will be better able to fulfill their obligations in the reciprocal relationship and realize the value of their own existence in the team by helping others, which brings about a positive impact on others and the organization. Perceived coworker support is a process of internalizing the external environment, such as team interpersonal relationships, through one’s own cognitive mechanisms. Such a self-identification process promotes the continuation and development of positive behaviors.

In short, this study advances OCB-H theory beyond static antecedents and time-lagged associations by establishing a new dynamic, peer-driven internalization model of sustained helping behavior.

### Practical implications

At the practical level, this paper provides guidelines for internal team management and coworker relationship management. In recent years, large amount of research focus on the relationship between leaders and members, or different styles of leadership (e.g., abusive management, transformational leadership, caring leadership style, etc.). This study starts from another angle of team social relationship and shifts from the perspective of ‘one-to-many’ to ‘many-to-many’. In the modern workplace, both organizations and individuals would like to have a harmonious and cohesive team. We prefer to be in contact with colleagues who are caring and appreciative of our abilities. We reject a selfish, snobbish, and indifferent workplace atmosphere. Mutual help among team colleagues, good reciprocal relationships, and timely and effective feedback on others’ ideas and opinions. These are the keys for employees to maintain interpersonal citizenship behavior. From the individual’s point of view, the willingness to give consistently to team members depends on one’s perceived interpersonal relationships. Such interpersonal relationships also constitute a circular effect with one’s own behavior, demonstrating the science of ‘Do not do unto others what you would not have them do unto you’. From the perspective of managers, this study will help them understand the impact of internal interpersonal relationships on team and individual performance. Moreover, how to encourage employees to produce positive performance and coordinate employee relations in daily management will become the focus of practical attention.

### Limitations and future research directions

This paper has the following limitations.

First, sample context and generalizability: the source sample of the data comes from the same province, which only focuses on the influence of employees’ perceived interpersonal relationships on the persistence of OCB-H. It fails to analyze and consider the influence of the nature of the organization, the work, and employees’ personalities. The results are inconsistent, which means that whether the research findings can be replicated to employees in other nations, enterprises or industries needs further verification.

Second, self-report data: all variables were measured using self-reported data, which introduces the potential for common method variance. Despite our efforts to mitigate this issue through the use of CFA model comparison and Harman’s test, these methods have limitations. Harman’s test, in particular, is a relatively weak diagnostic tool for detecting common method bias. Therefore, the presence of common method bias remains a limitation, and results should be interpreted with caution. Future research could incorporate multi-source data, such as supervisor or peer ratings, to enhance the robustness of the findings and reduce the impact of common method bias.

Third, short time lag: we employed a two-wave longitudinal design with a one-month interval between measurements. While this design supports temporal ordering to some extent, it does not allow for strong causal inference. The relatively short time lag between measurements may not fully capture the dynamic processes underlying the relationships we studied, particularly the internalization of interpersonal relationship perceptions into behavioral motivation. Future research should consider longer time intervals or multiple measurement points to better understand the temporal dynamics of these relationships.

Fourth, incomplete temporal separation of mediation: in our study, perceived coworker support and Phase-2 OCB-H were measured at the same time point, which means that the mediation process is not fully temporally separated. This limitation hinders our ability to make definitive causal claims about the mediating role of perceived coworker support in the relationship between perceived team-member exchange and OCB-H persistence. Future research should aim to measure mediators and outcomes at distinct time points to strengthen causal inferences.

Fifth, theoretical deduction and process mechanism: given that the process mechanism of internalization of employees’ external environment is not the primary focus of this study, our theoretical deduction is relatively rough and not meticulous, especially concerning the process of emotional cognition and attitude transformation. Future research should delve deeper into these processes, possibly through in-depth interviews and experiments, to better understand how interpersonal interactions influence employees’ psychological and behavioral changes.

Sixth, scale breadth: The 5-item scale of OCB-H, while concise, may lack the breadth needed to fully capture the multifaceted nature of helping behaviors. More items could potentially improve reliability by covering a wider range of helping behaviors, such as task-related help, emotional support, and informational support. Future research should consider refining the OCB-H scale to ensure greater contextual relevance and improve its psychometric properties.

Despite these limitations, our study contributes to the existing literature by exploring the continuity of OCB-H from a dynamic perspective and examining the effects of perceived team-member exchange relationships and coworker support. We provide universal suggestions and references for individuals and managers to deal with interpersonal relationships and coordinate their own behaviors. Future research can build upon our findings to further enrich the framework and address the limitations identified herein.

## Data Availability

The raw data supporting the conclusions of this article will be made available by the authors, without undue reservation.
